# Implications of Butyrate Signaling Pathways on the Motor Symptomatology of Parkinson’s Disease and Neuroprotective Effects—Therapeutic Approaches: A Systematic Review

**DOI:** 10.3390/ijms25168998

**Published:** 2024-08-19

**Authors:** Jorge Missiego-Beltrán, Eva María Olalla-Álvarez, Ana González-Brugera, Ana Isabel Beltrán-Velasco

**Affiliations:** NBC Group, Psychology Department, School of Life and Nature Sciences, Nebrija University, 28015 Madrid, Spain; jmissiegob@alumnos.nebrija.es (J.M.-B.); eolallaa@alumnos.nebrija.es (E.M.O.-Á.); agonzalezb21@alumnos.nebrija.es (A.G.-B.)

**Keywords:** butyrate, Parkinson’s Disease, motor symptomatology, neurodegenerative disease, gut microbiota, therapeutic approaches

## Abstract

Parkinson’s Disease (PD) is a prevalent neurodegenerative disorder characterized by motor and non-motor symptoms. Emerging evidence suggests that gut microbiota alterations, specifically involving short-chain fatty acids (SCFAs) like butyrate, may influence PD pathogenesis and symptomatology. This Systematic Review aims to synthesize current research on the role of butyrate in modulating motor symptoms and its neuroprotective effects in PD, providing insights into potential therapeutic approaches. A systematic literature search was conducted in April 2024 across databases, including ScienceDirect, Scopus, Wiley, and Web of Science, for studies published between 2000 and 2024. Keywords used were “neuroprotective effects AND butyrate AND (Parkinson disease OR motor symptoms)”. Four authors independently screened titles, abstracts, and full texts, applying inclusion criteria focused on studies investigating butyrate regulation and PD motor symptoms. A total of 1377 articles were identified, with 40 selected for full-text review and 14 studies meeting the inclusion criteria. Data extraction was performed on the study population, PD models, methodology, intervention details, and outcomes. Quality assessment using the SYRCLE RoB tool highlighted variability in study quality, with some biases noted in allocation concealment and blinding. Findings indicate that butyrate regulation has a significant impact on improving motor symptoms and offers neuroprotective benefits in PD models. The therapeutic modulation of gut microbiota to enhance butyrate levels presents a promising strategy for PD symptom management.

## 1. Introduction

### 1.1. Parkinson Disease

Currently, Parkinson’s Disease (PD) is one of the most prevalent neurodegenerative diseases worldwide, second only to Alzheimer’s disease. It is estimated that almost 7 million individuals currently suffer from this pathology, with an increase of up to 12 million predicted by the year 2040 [1]. Despite extensive research, the etiology of PD remains unknown [2]. However, studies in this field suggest a combination of genetic and environmental elements may play a role [3,4].

Although it is a sporadic disease with no known genetic cause, a percentage of between 5 and 10% of cases have been associated with a family origin, identifying certain genes that cause PD [5]. Specifically, more than 20 loci and 19 genes causing this pathology have been identified, although the genes of certain PARK loci have not yet been recognized in this pathology [6,7]. In this regard, the *LRRK2* gene may account for the largest number of cases [8,9].

In terms of pathological anatomy, it is well-established that PD is characterized by a progressive loss of dopaminergic neurons in the substantia nigra pars compacta of the midbrain [10]. Additionally, Lewy bodies, which are an agglomeration of abnormally folded alpha-synuclein (αSyn) proteins, can be observed in the brainstem, which is associated with the articulation of movements [11,12]. If this accumulation extends to other, outermost layers of the brain, individuals with PD may also present dementia-associated symptomatology [13]. This results in a physiological alteration of the basal ganglia, which explains the manifestation of the symptoms of the disease [14].

The selective death of dopaminergic neurons of the substantia nigra pars compacta is the primary mechanism underlying the clinical features of PD [15,16]. This primarily manifests as motor symptoms, including resting tremor, rigidity, and bradykinesia. As the disease progresses, the motor symptoms become more pronounced, accompanied by gait difficulty, postural instability, and dysphagia [17,18]. It is also important to note that there are a number of non-motor symptoms that complicate the pathogenesis of the disease. These include psychotic symptoms, digestive problems, and mood disorders, among others [19,20].

With regard to the control of movement, it is now understood that the motor symptoms that are characteristic of PD are explained by an altered functioning of the circuitry that is involved in the control of movement in the brain. This circuitry is composed of the basal ganglia, including the striatum and the internal and external globus pallidus, as well as other associated nuclei such as the subthalamic nucleus and the substantia nigra pars reticulata [21,22,23].

### 1.2. Butyrate Signaling Pathways in PD

PD has been traditionally studied from the mitochondrial alterations presented in these patients. These alterations include an increase in αSyn, dysfunction of the electron transport chain complex, and mechanisms of parkinsonism, which is regulated by MPTP (1-methyl-phenyl,6-tetrahydropyridine) [24]. These organic dysfunctions have been related to alterations in the Enteric Nervous System (ENS) and the parasympathetic branch of the Autonomic Nervous System (ANS) [25,26].

Recent studies have demonstrated that bacterial families and specific metabolites produced in the intestine exert a significant influence on the pathogenesis of PD [27,28]. This association has been observed in patients presenting with dysbiosis, a condition characterized by a proinflammatory intestinal state [29]. It is now evident that dysbiosis plays a relevant role in the development of the disease through the bidirectional communication of the microbiota–gut–brain axis [30,31,32].

In particular, short-chain fatty acids (SCFAs) or volatile fatty acids (VFAs) constitute a subgroup of fatty acids with carbon chains of less than six carbon atoms [33]. SCFA are produced in the gastrointestinal tract, specifically in the colon, and the main ones are acetic acid or acetate, propionic acid or propionate, and butyric acid or butyrate [34,35]. Butyrate has been associated with trophic, nutritional, and anti-inflammatory effects on the intestinal epithelium. Consequently, it plays a pivotal role in maintaining the intestinal mucosa and epithelium. In this line, several studies have demonstrated that *Roseburia*, *Faecalibacterium*, and *Anaerostipes* are correlated with butyrate abundance, among others [36,37].

These fatty acids are essential signaling molecules in the stimulation of neurotransmitters in the Central Nervous System (CNS), in addition to inhibiting apoptosis [38]. They access the brain by crossing the blood–brain barrier (BBB) through free fatty acid receptors. In this context, recent studies have demonstrated the presence of acetate, propionate, and butyrate in human cerebrospinal fluid (CSF), with concentrations of 0–171 μM for acetate, 0–6 μM for propionate, and 0–2.8 μM for butyrate [39,40,41].

It has been established that patients with PD present a significant reduction in butyrate-synthesizing bacterial genera, including *Bacteroides*, *Prevotellaceae*, *Faecalibacterium prausnitzii*, *Lactobacillaceae*, and *Enterococcaceae*. This reduction in bacterial diversity leads to a reduction in acetate, propionate, and butyrate concentrations compared to control groups [42,43]. Furthermore, these patients frequently exhibit elevated levels of pro-inflammatory bacterial species, including *Odoribacter splanchnicus* and *Bacteroides vulgatus*, among others [44,45].

Recent studies have demonstrated that butyric acid is a potent inhibitor of histone deacetylase (HDAC), which improves cognitive functioning associated with neurodegenerative diseases and is involved in cell signaling processes, impacting intracellular K^+^ levels [37,46]. Additionally, SFCA modulates the expression of the gene encoding the enzyme essential in serotonin biosynthesis (tryptophan hydroxylase), which is involved in neuronal regeneration and survival [47,48].

Data on the effects of modulation of the gut microbiota, specifically the regulation of butyrate levels, appear to be promising for the intervention of motor symptoms in Parkinson’s Disease. In the last few years, research in this area has increased significantly and needs to be thoroughly analyzed.

The main objective of this Systematic Review was to collect all significant findings from animal model studies on the impact of butyrate regulation on the improvement in motor symptomatology and its neuroprotective effects in Parkinson’s Disease. This study will provide the most current data on the use of this significant metabolite, thereby facilitating future research and intervention initiatives in this field of study.

## 2. Results

**Figure 1:**
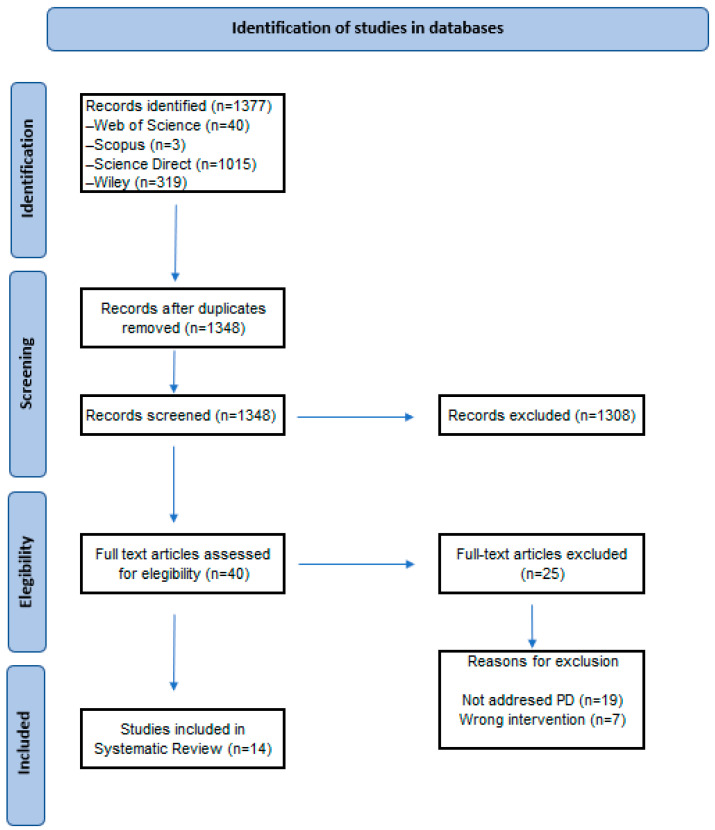
A PRISMA flow diagram of the search and selection process.

A total of 1377 articles were retrieved, of which 40 were sought for full-text review. The full titles and abstracts of all articles were examined for eligibility. Following full-text checking, a total of 14 studies were included in this article. All the selected articles were retrieved for comprehensive review, based on the established inclusion criteria, as shown in the PRISMA diagram (Figure 1).

### 2.1. Data Extraction

Study data were extracted into a customized spreadsheet by four reviewers (J.M.-B., E.M.O.-A., A.B.-G., and A.I.B.-V.). The data extracted from the included studies were as described below (Table 1):

- Population (description).
- Model PD-induced.
- Intervention (dose administered, time, and frequency).
- Methodology and results obtained after the intervention.

**Table 1:** Implications of butyrate signaling pathways on the motor symptomatology of Parkinson’s Disease (PD).

Ref.	Population and Model	Intervention	Methodology and Results
[49]	PD male Sprague–Dawley rats induced by 6-OHDA (12 μg in 0.1% ascorbic acid)	The sham-injured animals were administered an ascorbic acid/acidic saline solution (4 mL). Prior to induction of injury, MPEP or L-AP4 (2 nmol in 4 mL) were administered intraneural, alone or in combination. The controls received the vehicle alone, and the treatment was continued for 7 days	Sham and control groups were observed
			Significant attenuation of nigral TH-IR cell loss with either MPEP or L-AP4 alone
			Greater preservation of nigral TH-IR cells with coadministration
			Significant attenuation of DA and metabolite depletion with either MPEP or L-AP4 alone
[50]	PD C57BL/6 mice (4–6 months old) induced by MPTP (20 mg/kg)	The control mice were administered water containing equimolar concentrations of sodium chloride, PB (phenylbutyrate), and NaB for a period of 14 days. The PB and NaB groups were administered these substances in water at concentrations of 500, 1000, 1500, and 2000 mg/L	The control group, PB group, and NaB group were observed
			Notable elevation in DJ-1 levels
			No change in α-synuclein concentrations, suggesting a selective increase in DJ-1 expression
			PB treatment resulted in a significantly higher number of TH-positive dopaminergic neurons in the substantia nigra
[51]	PD adult male Wistar rats induced by 6-OHDA (8 μg in 1 μL in 0.2% ascorbic acid)	A single stereotaxic dose of 1 μL of 5-HT (10 μg/μL), GABA (10 μg/μL), and BMC (10^6^ cells/μL) in combinations was infused (day 18) into the right SNpc (0.2 μL/min)	Control, 6-OHDA, (6-OHDA + BMC), (6-OHDA + 5-HT + BMC), (6-OHDA + GABA + BMC), and (6-OHDA + 5-HT + GABA + BMC) were observed
			The combinational treatment of 5-HT, GABA, and BMC resulted in a significant attenuation of TBAR levels
			Significant reversal of SOD, CAT, and GPx enzyme activities to near-control levels
[52]	PD male C57BL/6 mice (6–8 weeks old) induced by MPTP (30 mg/kg)	Sham group (intraperitoneal injection of normal saline). NaB (200 or 600 mg/kg) gavage-treated groups (3 weeks)	Sham group, MPTP group, MPTP + NaB group (200 mg/kg), and MPTP + NaB group (600 mg/kg) were observed
			NaB relieved MPTP-triggered motor dysfunction and dopaminergic neuronal death in mice
			Mitigated MPP^+^-induced apoptosis
			NaB alleviated MPP^+^-stimulated oxidative stress and inflammatory responses in PC12 cells
[53]	PD male C57BL mice (8–10-weeks-old) induced by MPTP (18 mg/kg)	Vehicle, DβHB (1.6, 0.8, or 0.4 mmol/kg/day in saline, pH 7.4), and LβHB (1.6 mmol/kg/day in saline, pH 7.4) were administered subcutaneously (1 μL/h). 3-NP (15 mg/kg in 0.1 M PBS adjusted to pH 7.4) was administered intraperitoneally 2 h before implantation on day 1.	The vehicle group, DβHB group, LβHB group, and DβHB plus 3-nitropropionic acid (3-NP) groups were observed
			DβHB has shown significant improvement in motor function
			Complete restoration of MPP^+^-inhibited oxygen consumption is only partially possible in the presence of rotenone
			No antioxidant effects
			Enhanced ATP production
[54]	PD male C57BL/B6 mice induced by MPTP (30 mg/kg)	A solution of NaB diluted in normal saline (200 mg/kg) was administered intragastrically for 3 weeks after a 7-day treatment with MPTP. Groups (1) and (2) received an equal volume of saline	Control, PD model, and NaB treatment groups were observed
			NaB treatment has shown an effective alleviation of motor deficits and an improved state of despair in PD model mice
			Significant elevation in TH expression
			Statistically higher number of TH-positive neurons in the substantia nigra compared to the PD group
[55]	PD male C57BL/6 mice (6–8 weeks old) induced by Mn (30 mg/kg)	An intraperitoneal injection of VPA (200 mg/kg), NaB (1200 mg/kg), or saline (NaCl, 0.9%; control) was administered for 21 days. VPA and NaB were diluted in saline (0.9%). After a 30 min period, the Mn + VPA, Mn+NaB, and Mn groups received 2 μL of MnCl_2_ (30 mg/kg)	The control group, VPA group, Mn group, Mn plus VPA group, NaB group, and Mn plus NaB group were observed
			Significant reversal of Mn-induced motor deficits by both VPA and NaB
			No change in rotarod performance with VPA or NaB alone compared to control
			NaB co-treatment with Mn attenuated Mn-decreased GLT-1 mRNA levels in the cerebral cortex and cerebellum, similar to VPA
			NaB reversed the Mn-induced reduction of GLAST mRNA levels in the cortex
[56]	PD male C57BL/6J mice (7-weeks-old) induced by MPTP (15 mg/kg)	The probiotic group consumed approximately 2 × 10^6^ CFU of microorganisms/day for 30 days (*Lactobacillus rhamnosus* GG, *Bifidobacterium animalis* lactis, and *Lactobacillus acidophilus*; vehicle (lactose+maltodextrin)	First, mice were divided into vehicle- and probiotic-treated groups, which were then subdivided into saline- and MPTP-administered groups
			Probiotics have shown a reduction in motor errors induced by MPTP
			Significant prevention of dopaminergic nerve terminal loss in the striatum
			Attenuation of MPTP-mediated astrocyte activation
			Significant prevention of BDNF and GDNF suppression in substantia nigra tissue
[57]	PD male C57BL/6J mice (8-week-old) induced by MPTP (15 mg/kg)	The control group received a daily oral gavage of distilled water (4 weeks) followed by a saline injection. The PD group received distilled water by oral gavage (4 weeks), followed by an injection of MPTP. The polymannuronic acid-treated group received polymannuronic acid (30 mg/kg) by oral gavage (4 weeks)	Normal, model, and PM groups were observed
			PM treatment has shown a notable improvement in motor functions
			Substantial elevation of HVA, 5-HT, and 5-HIAA levels in the striatum
			Significant increase in fecal concentrations of total SCFAs and specific SCFAs (acetic acid, propionic acid, and butyric acid), contributing to neuroprotective effects
[58]	PD male C57BL/6J mice (8-weeks-old) induced by MPTP (30 mg/kg)	PD mouse group (0.2 g/kg or 2.0 g/kg sodium acetate-NaA-L or NaA–H), PD mouse group (0.2 g/kg or 2.0 g/kg of sodium propionate-NaP-L or NaP-H), group of PD mice (0.2 g/kg or 2.0 g/kg of sodium butyrate-NaB-L or NaB-H), and group of PD mice (0.1 g/kg levodopa as positive control group (L-dopa)	(Con), (PD), (NaA-L or NaA–H), (NaP-L or NaP-H), (NaB-L or NaB-H), and (L-dopa) groups were observed
			Only NaB treatment has shown a notable enhancement in motor functions
			NaB was identified as the most efficacious in mitigating brain damage
			Discernible increase in dopaminergic positive neuronal cells and reduction in α-synuclein accumulation
[59]	PD male Swiss CD1 mice (10-week-old) induced by 6-OHDA (4 µg/2 µL)	Sham control group (intrastriatal injection of vehicle); 6-OHDA, 6-OHDA+NaB (6-OHDA and NaB treated); 6-OHDA + CFX, (CFX for 5 days and intrastriatal injection of 6-OHDA); 6-OHDA + CFX + NaB (CFX, intrastriatal injection of 6-OHDA and NaB treatment); and CFX (antibiotic solely for 5 days)	Sham control mice, 6-OHDA, 6-OHDA + NaB, 6-OHDA + CFX, 6-OHDA + CFX + NaB, and CFX groups were observed
			NaB (in 6-OHDA and dual-insulted mice) showed improvement in motor coordination by day 7
			Increased Bcl-2 immunoreactivity and decreased Bax expression in the striata
			Reversal of the Bcl-2/Bax ratio in untreated 6-OHDA and 6-OHDA + CFX mice
			Limited systemic inflammation and endotoxemia in 6-OHDA + CFX mice
[60]	PD male C57BL/6J mice (7-weeks-old) induced by MPTP (20 mg/kg)	In the second and third weeks, in the MPTP + NaB group and the MPTP + MMF group (NaB: 600 mg/kg/day) and (MMF: 100 mg/kg/day), PBS (10 mL/kg/day) was administered to both the control group and the MPTP + vehicle group.	The control group, MPTP + vehicle group, MPTP + NaB group, and MPTP + MMF group were observed
			NaB and MMF showed alleviated coordination impairment compared to the control group
			Reduced loss of TH-positive dopaminergic neurons in the SNpc
			Substantially reduced serum levels of IL-6 and TNF-α
[61]	PD male C57BL/6J mice (7-weeks-old) induced by MPTP (30 mg/kg)	MPTP and MPTP + NaB groups (30 mg/kg MPTP intraperitoneally) for 7 days. Control group (normal saline); NaB or saline intragastrically (14 days) after a 2 h interval after the 21-day MPTP injection period	The control group, MPTP group, and MPTP + NaB group were observed
			NaB treatment has shown significant improvement in motor functions
			A marked increase in DA and 5-HT levels in the striatum compared to MPTP mice
			Reduction in GFAP expression and suppression of glial cell activation, leading to reduced neuroinflammation
[62]	PD male C57BL/6 mice (8-week-old) induced by MPTP (30 mg/kg)	Group C (saline gelatin—14 days); Group M (saline gelatin—7 days); Group L (intraperitoneal injection of 0.4 mg/kg liraglutide—7 days); Group CB (10^8^ CFU. of *C. butyricum* by gavage in saline containing 0.01% gelatin—7 days); Group CBG (same dose and duration)	G, C, M, L, CB, and CBG groups were observed
			Significant enhancement of locomotor capacity in the M group *C. butyricum*-GLP-1 or liraglutide treatment
			No statistical difference between *C. butyricum*-GLP-1 and liraglutide treatments

### 2.2. Quality of Included Studies

The RoB tool for animal intervention studies (SYRCLE RoB tool) is based on the Cochrane RoB tool, with specific adjustments made to account for biases that are particularly relevant in animal intervention studies [49].

**Figure 2:**
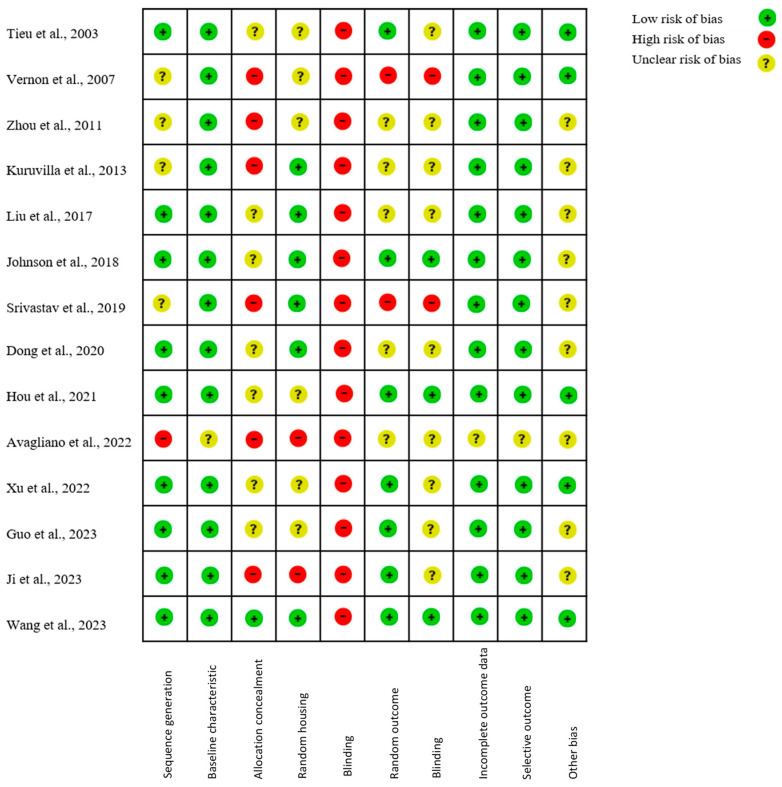
SYRCLE’s tool for assessing the risk of bias [49,50,51,52,53,54,55,56,57,58,59,60,61,62,63].

The results of the quality assessment are presented in Figure 2. The quality of the studies varies considerably depending on the risk of bias category. While some aspects, such as sequence generation and handling of incomplete outcome data, are well-reported and show a low risk of bias, other areas, such as allocation concealment, blinding, and random housing, often lack clarity or present a high risk of bias. 

### 2.3. Neuroprotective Effects of Butyrate in PD

In 2007, Vernon, Chroucher, and Dexter conducted a study using a 6-hydroxydopamine (6-OHDA)-induced PD model. L-(+)-2-amino-4-phosphonobutyric acid (L-AP4) and/or 2-methyl-6-(phenylethyl) pyridine (MPEP) were administered to male Sprague-Dawley rats to assess their neuroprotective effects. The results demonstrated that L-AP4 exhibited a markedly enhanced neuroprotective impact. Intranigral administration of MPEP or L-AP4 (2 nmol) independently led to a notable reduction in dopamine (DA) and its metabolites. The concomitant administration of MPEP and L-AP4 also markedly prevented striatal DA depletion, with a discernible trend towards greater protection compared to MPEP or L-AP4 alone. However, this did not reach statistical significance [49].

In 2011, Zhou et al. conducted a study using a 1-methyl-4-phenyl-1,2,3,6-tetrahydropyridine (MPTP)-induced PD model in C57BL/6 mice to evaluate the impact of phenylbutyrate and NaB (sodium butyrate) on neuronal protection in this pathology. The results demonstrated that both treatments markedly elevated DJ-1 levels in comparison to the control group. Furthermore, α-Synuclein concentrations remained unaltered following drug administration, indicating selective induction of increased DJ-1 expression in the brain. Additionally, mice treated with phenylbutyrate exhibited a significantly enhanced number of TH-positive dopaminergic neurons in the substantia nigra [50].

In the study by Kuruvilla, Nandhu, and Paulose (2013), the neuroprotective potential of gamma-aminobutyric acid (GABA) was evaluated in a mouse model of PD based on 6-OHDA using male Wistar rats. The findings demonstrated that the concurrent administration of 5-HT, GABA, and BMC markedly reduced the concentration of TBAR (thiobarbituric acid reactive substances) in comparison to the 6-OHDA-treated rats. Moreover, the combined treatment led to a notable restoration of SOD (superoxide dismutase), CAT (catalase), and GPx (glutathione peroxidase) enzyme activities, approaching the levels observed in the control group [51].

In the study by Ji et al. (2023), the impact of NaB on the inhibition of the JAK2/STAT3 signaling pathway was evaluated to determine its efficacy in reducing MPP^+^/MPTP-induced neurotoxicity. Male C57BL/6 mice were administered NaB (200 or 600 mg/kg) via gavage after the induction of MPTP. The results indicated that NaB alleviated MPTP-induced motor dysfunction and dopaminergic neuronal death. Specifically, the NaB-treated group (200 mg/kg) demonstrated a statistically significant reduction in apoptosis, while the NaB-treated group (600 mg/kg) exhibited a highly significant reduction. Furthermore, NaB mitigated MPP^+^-induced apoptosis and alleviated oxidative stress and inflammatory responses in PC12 cells [52].

In summary, the studies reviewed highlight the potential of various neuroprotective agents in mitigating the pathophysiological processes underlying PD. L-AP4 exhibited a markedly augmented neuroprotective impact in 6-OHDA-induced PD models, with concomitant administration of L-AP4 and MPEP displaying a tendency towards augmented protection against dopamine depletion. The administration of phenylbutyrate and NaB has been demonstrated to significantly elevate DJ-1 levels and preserve dopaminergic neurons in the substantia nigra, thereby highlighting the selective neuroprotective mechanisms of these agents without altering α-synuclein levels. Moreover, the combination of 5-HT, GABA, and BMC effectively reduced oxidative stress markers and restored antioxidant enzyme activities. Additionally, NaB was found to inhibit the JAK2/STAT3 signaling pathway, thereby reducing apoptosis, oxidative stress, and inflammation in PD models. These findings collectively indicate that a promising therapeutic strategy for PD may be to target oxidative stress, inflammation, and apoptotic pathways through modulation of the gut microbiota.

### 2.4. Efficacy of Butyrate Regulation in Improving Motor Symptoms in PD

In 2003, Tieu and colleagues conducted a study to address the impact of D-β-hydroxybutyrate (DβHB) in preventing neuronal damage produced by PD and to elucidate the motor symptomatology of the disease. The study utilized C57BL MPTP-induced mice, and they were treated with DβHB subcutaneously at a rate of 1 μL/h. The results indicated that the induced motor deficits were significantly improved with DβHB treatment compared to the other groups. Additionally, DβHB restored MPP^+^-inhibited oxygen consumption to its original level but was only partially inhibited by rotenone. It is noteworthy that DβHB has no antioxidant effects but increases ATP production [53].

In the study by Liu et al. (2017), the protective effects of NaB were analyzed in a C57BL/B6 murine model of MPTP-induced PD. The NaB group received a dose of 200 mg/kg administered intragastrically after a seven-day period during which MPTP was administered. The administration of NaB resulted in a notable reduction in the MPTP-induced motor deficit. Furthermore, NaB treatment was observed to alleviate the despair observed in PD model mice, and the expression of TH was significantly elevated in comparison to both PD groups. The number of remaining TH-positive neurons in the SN demonstrated a statistically higher count than in the PD group [54].

In their 2017 study, Johnson and colleagues investigated the potential of valproate and NaB to mitigate locomotor deficits in an animal model of Mn-manganese-induced neurotoxicity. C57BL/6 mice were administered a single intraperitoneal injection of either VPA (200 mg/kg) or NaB (1200 mg/kg). The findings indicated that the Mn-induced reduction in total distance traveled was markedly reversed by both VPA and NaB. The VPA and NaB alone groups did not demonstrate any alterations in rotarod performance when compared to the control group. The combined treatment of NaB with Mn demonstrated a comparable effect to VPA in mitigating the Mn-induced reduction in GLT-1 mRNA levels in the cerebral cortex and cerebellum. Furthermore, NaB was observed to reverse the Mn-induced reduction in GLAST (glutamate aspartate transporter) mRNA levels in the cortex, with a statistically significant effect [55].

In a study conducted by Srivastav et al. (2019), the efficacy of a probiotic treatment was evaluated in a C57BL/6J mouse model of PD induced by MPTP. The probiotic group received a mixture containing approximately 2 × 10⁶ colony-forming units (CFU) of microorganisms per day. Probiotic supplementation appeared to reduce the number of motor errors induced by MPTP. Furthermore, probiotic pretreatment was found to attenuate MPTP-mediated astrocyte activation and significantly prevent the suppression of BDNF (brain-derived neurotrophic factor) and GDNF (glial cell-derived neurotrophic factor) in the substantia nigra [56].

The study by Dong et al. (2020) evaluated the impact of polymannuronic acid (PM) in a mouse model of MPTP-induced PD in male C57BL/6J mice. The findings indicated that PM treatment markedly enhanced motor functions in mice with PD and elicited a notable elevation in HVA levels. The results demonstrated a notable elevation in the levels of 5-HT, 5-HIAA (a metabolite of 5-HT), and GABA in the striatum of PD mice when compared to the control group. Furthermore, the oral administration of MP was observed to significantly elevate the fecal concentrations of total SCFA, as well as all three measured SCFA, including acetic acid, propionic acid, and butyric acid, in PD mice [57].

In a study conducted in 2021, Hou and colleagues evaluated the neuroprotective effect of SCFA in a model of male C57BL/6J mice with MPTP-induced PD. The groups of mice with PD were treated with 0.2 g/kg or 2.0 g/kg sodium acetate, 0.2 g/kg or 2.0 g sodium propionate, or with 0.2 g/kg or 2.0 g/kg sodium butyrate. The findings demonstrated that, among the three types of SCFA, only NaB exhibited a notable improvement in motor deficits. Additionally, across all three types of SCFA, NaB was identified as the most efficacious substance for mitigating brain damage. Both low and high concentrations of NaB were observed to significantly enhance the proportion of dopaminergic positive neuronal cells and reduce the accumulation of α-synuclein in PD mice [58].

The study by Avagliano et al. (2022) examined the neuroprotective and anti-inflammatory effects of NaB in a male Swiss CD1 mouse model with 6-OHDA-induced PD. The findings indicated that NaB treatment resulted in enhanced motor coordination over time in both 6-OHDA and dual-insulted mice. The immunoreactivity for Bcl-2 increased significantly, while Bax expression decreased significantly in the striatum of NaB-treated groups. This resulted in a reversal of the reduced ratio observed in the untreated 6-OHDA or 6-OHDA + CFX mice. Furthermore, NaB administration was found to have a limiting effect on the development of systemic inflammation and endotoxemia in mice subjected to a combination of 6-OHDA and CFX [59].

A recent study by Xu et al. (2022) examined the neuroprotective and motor impairment effects of NaB and MMF-monomethyl fumarate in a male C57BL/6J mouse model of MPTP-induced PD. The findings indicated that NaB and MMF can mitigate coordination deficits in mice with PD. Furthermore, the number of TH-positive dopaminergic neurons in the pars compacta of the substantia nigra (SNpc) was markedly diminished in the MPTP + vehicle group relative to the control group. Conversely, treatment with NaB and MMF resulted in a significant reduction in the serum levels of IL-6 and TNF-α [60].

Guo et al. (2023) conducted a study to evaluate the neuroprotective effects of NaB in a male C57BL/6 mouse model of MPTP-induced PD. The findings of this study indicated that mice treated with MPTP and NaB demonstrated notably enhanced performance in motor assessments, including the pole and the rotarod test. Furthermore, the study revealed a notable elevation in DA levels in the striatum, with a 49.2% increase observed in MPTP + NaB mice compared to MPTP mice. Furthermore, 5-HT levels were observed to be reduced by 34.6% in MPTP-treated mice, while they demonstrated a 49.2% increase in MPTP + NaB mice. NaB treatment resulted in a 26.2% reduction in GFAP expression in MPTP-treated mice, accompanied by a notable inhibition of glial cell activation and a subsequent reduction in neuroinflammation [61].

A recent study by Wang et al. (2023) evaluated the impact of the probiotic *Clostridium butyricum* pMTL007-GLP-1 in a male C57BL/6 mouse model of PD induced by MPTP. A dosage of 10^8^ CFU/mL was administered. The results demonstrated that the locomotor ability observed in group M was markedly enhanced by treatment with *C. butyricum*-GLP-1 or liraglutide. The mean time to complete the task was 272 s in the liraglutide treatment group (L) and 10.87 ± 1.708 s in the control group (M), with a statistically significant difference. No statistically significant difference was observed between *C. butyricum*-GLP-1 and liraglutide [62].

A synthesis of the findings of the reviewed studies indicates that various compounds have the potential to offer neuroprotective and motor function-enhancing benefits in different models of PD. DβHB was observed to significantly improve motor deficits and restore oxygen consumption, despite the absence of antioxidant properties. NaB emerged as a particularly efficacious agent, demonstrating consistent efficacy in reducing motor deficits, enhancing dopaminergic neuron survival, and mitigating neuroinflammation across multiple studies. Furthermore, NaB demonstrated the capacity to reverse the neurotoxic effects of MPTP and Mn and exhibited greater efficacy than other short-chain fatty acids in reducing brain damage and α-synuclein accumulation. Furthermore, probiotics and polymannuronic acid were demonstrated to enhance motor functions and exert neuroprotective effects, thereby reinforcing the therapeutic potential of gut–brain axis modulation in PD. Collectively, these findings highlight the potential of metabolic and anti-inflammatory approaches in the treatment of PD.

## 3. Discussion

The main objective of this Systematic Review was to collect all significant findings from animal model studies on the impact of butyrate regulation on the improvement in motor symptomatology and its neuroprotective effects in Parkinson’s Disease. The results obtained indicate that the regulation of butyrate levels has a neuroprotective effect in PD and significantly improves the motor impairment associated with this pathology.

This study synthesizes findings from preclinical investigations that underscore the therapeutic potential of various compounds in mitigating the pathological processes underlying PD. The results of these studies consistently demonstrate that agents that target oxidative stress, inflammation, and metabolic dysregulation have significant neuroprotective effects, which are crucial factors in the pathogenesis of PD. Thus, DβHB has been demonstrated to markedly improve motor deficits in PD models, restore mitochondrial function by reversing MPP^+^-inhibited oxygen consumption, and enhance ATP production. However, its efficacy is reduced in the presence of rotenone, indicating that while DβHB effectively targets metabolic dysfunction, it may not be a sufficient standalone intervention in environments of complex oxidative stress. This is consistent with the observation that DβHB lacks antioxidant effects. In this line, previous studies have investigated the potential neuroprotective effects of SCFAs. A recent study by Liu et al. (2021) demonstrated that the administration of acetate (OAc), propionate (Pr), and NaB can significantly improve neuronal degeneration [63]. Similarly, Huang et al. (2017) observed that SCFAs interact with key factors in this pathology, such as reducing oxidative stress and inflammatory responses in the organism [64].

Sodium butyrate has emerged as a particularly efficacious neuroprotective agent. The results of numerous studies indicate that NaB consistently alleviates motor deficits, enhances the survival of dopaminergic neurons in the substantia nigra, and reduces neuroinflammation. These effects were observed in multiple models of PD, including those induced by MPTP, manganese-Mn, and 6-OHDA. It is noteworthy that NaB has the capacity to reverse apoptotic markers and reduce oxidative stress, which illustrates the extensive mechanism of action that it employs. Furthermore, its efficacy in enhancing dopaminergic neuron survival and reducing α-synuclein accumulation serves to identify NaB as a promising candidate for further investigation in the field of PD therapy.

Previous studies, such as the one conducted by Lanza et al. in 2019, have confirmed the efficacy of NaB in reducing oxidative stress and reversing apoptotic markers. This evidence supports the assertion that NaB has a broad mechanism of action in neuroprotection. Additionally, modulation of gut–brain axis inflammation is a significant aspect of NaB efficacy, as evidenced by its ability to restore intestinal microbial balance and prevent systemic inflammation. This is increasingly recognized as a contributing factor to PD pathology [65]. 

Other studies that have addressed the neuroprotective role of SCFAs, primarily butyric acid, have demonstrated that the administration of distinct probiotic bacteria increases NaB levels, which has been shown to prevent the loss and depletion of dopaminergic neurons in the pars compacta of the substantia nigra. Srivastav et al. (2019) employed a combination of *Lactobacillus rhamnosus* GG, *Bifidobacterium animalis lactis*, and *Lactobacillus acidophilus* and were able to substantiate the neuroprotective and preventive effects on neurodegeneration associated with PD. Other studies have demonstrated the potential efficacy of probiotics and polymannuronic acid in the treatment of PD [56]. Administration of these agents has been shown to reduce astrocyte activation and preserve key neurotrophic factors, including brain-derived neurotrophic factor (BDNF) and glial cell-derived neurotrophic factor (GDNF), which are produced by glial cells. PM has been demonstrated to elevate the levels of neurotransmitters such as GABA and serotonin within the striatum of PD models while also increasing the levels of SCFAs within the intestine. This, in turn, has been shown to enhance the neuroprotective effects and regulate the bidirectional communication of the microbiota–gut–brain axis [57,66].

The combination of valproate and NaB has demonstrated considerable potential in mitigating manganese-induced neurotoxicity by restoring the expression of GLT-1 and GLAST mRNA, both of which are pivotal for maintaining glutamate homeostasis. This restoration is of vital importance since disruptions in glutamate transporters can lead to excitotoxicity, a process that plays a central role in neurodegeneration associated with PD. The ability of these compounds to normalize glutamate levels suggests a potential mechanism through which they may protect dopaminergic neurons from degeneration.

The findings of recent studies have reinforced those previously discussed, highlighting the broader implications of NaB and valproate in neuroprotection. For instance, research has demonstrated that NaB not only improves motor functions but also significantly reduces neuroinflammation, which is a common pathological feature in PD. These effects were particularly evident in models of manganese-induced neurotoxicity, where NaB’s impact on glutamate transporter expression directly correlated with its neuroprotective outcomes.

One of the principal mechanisms through which NaB exerts its neuroprotective effects is via the modulation of the JAK2/STAT3 signaling pathway, which plays a pivotal role in neuroinflammation and apoptosis. This pathway is especially pertinent in the context of PD, where chronic inflammation is a key contributor to the progressive loss of dopaminergic neurons. Prior research has demonstrated that NaB effectively inhibits the JAK2/STAT3 pathway, reducing both neuroinflammatory and apoptotic markers, which are of paramount importance in the pathogenesis of PD. Furthermore, NaB has been demonstrated to reinstate levels of GLT-1 and GLAST mRNA, which are crucial for maintaining glutamate homeostasis. This provides additional evidence to support the hypothesis that NaB can counteract excitotoxicity, which is a significant contributor to neurodegeneration in PD. This broader mechanism of action highlights NaB’s potential as a multifaceted therapeutic agent capable of addressing various pathogenic processes involved in PD, beyond just glutamate regulation [67,68,69].

Moreover, studies have examined the interactions between NaB and G-protein-coupled receptors (GPCRs), particularly GPR41 and GPR109A, which are involved in the anti-inflammatory and neuroprotective effects observed in PD models. This interaction further emphasizes the complex and multitargeted mechanism through which NaB exerts its beneficial effects, thereby making it a promising candidate for further research and development as a therapeutic agent in PD [70,71].

In regard to the motor symptomatology of PD and the impact of NaB on it, there is evidence to suggest that improvement can be observed. As previously stated, the implications of NaB on PD are profound. In this line, recent studies such as those by Elford et al. (2024) demonstrated that a reduction in butyrate levels can be a pivotal factor in the onset and progression of PD. Consequently, NaB may potentially alter the trajectory of this condition, which is associated with motor impairment [72]. Furthermore, Liu et al. and Elford et al. have demonstrated that NaB treatment reflects neuroprotective effects on dopaminergic neurodegeneration. Upon analysis of the treatment effects, a decrease in neuron loss was observed in comparison to the control group. This indicates a causal correlation between motor and dopamine impairment and suggests that NaB acts to reduce these manifestations and its neuroprotective potential [54,73,74,75].

These findings indicate that the administration of probiotics to regulate the intestinal microbiota may promote the production of essential SCFAs, such as butyrate, and potentially alleviate the motor symptoms of PD. Dong et al. (2024) investigated the impact of *Bifidobacterium animalis* subsp. lactis NJ241 in this context, demonstrating a notable reduction in MPTP-induced neuroinflammation [76].

Other studies have demonstrated that PM exerts a beneficial effect on motor functions in this pathology due to its anti-inflammatory properties and its capacity to protect CNS cells. Furthermore, the study conducted by Dong et al. (2020) demonstrated that the administration of PM or *Lactobacillus rhamnosus* GG, alone or in combination, has the potential to prevent the loss of dopaminergic neurons by enhancing the expression of the tyrosine hydroxylase gene and/or protein in the midbrain and striatum [57].

### 3.1. Limitations and Future Research

This study provides significant insights into the potential benefits of butyrate and probiotics in alleviating motor symptoms associated with Parkinson’s Disease (PD). However, several limitations must be acknowledged. First, the translation of these findings to clinical settings requires cautious interpretation and further validation through human clinical trials. 

In addition, the heterogeneity of PD pathology necessitates a broader investigation into how different strains of probiotics and varying doses of butyrate affect diverse patient populations. Future research should also investigate the long-term effects and safety profiles of these treatments, as the chronic nature of PD necessitates sustained therapeutic strategies. 

Another critical area for future research is to elucidate the precise mechanisms through which butyrate and probiotics exert their neuroprotective effects. Detailed molecular and cellular studies could identify the pathways involved, potentially leading to the identification of new therapeutic targets.

### 3.2. Practical Applications 

The use of butyrate, particularly sodium butyrate (NaB), and specific probiotic strains like *Bifidobacterium animalis* subsp. lactis NJ241, has been shown to have a beneficial effect on neuroinflammation and motor deficits. These interventions could be integrated into existing treatment regimens to enhance patient outcomes.

It is essential that clinicians consider recommending dietary adjustments or supplements that promote the production of butyrate and the maintenance of a balanced intestinal microbiota. Furthermore, the anti-inflammatory properties of polymannuronic acid (PM) suggest its potential as a complementary therapy for PD. Healthcare providers may wish to consider the use of PM as a preventive measure or as part of a comprehensive treatment plan aimed at reducing neuroinflammation and protecting dopaminergic neurons.

## 4. Methods

### 4.1. Protocol and Registration 

The Systematic Review protocol was submitted on 23 June 2024 (Identifier: osf-registrations-2hfaw-v1; Osf_project: https://api.osf.io/v2/nodes/7jgur/?version=2.20, (accessed on 23 June 2024)).

### 4.2. Literature Search

The review followed the Preferred Reporting Items for Systematic Reviews and Meta-Analyses (PRISMA) statement and the following previous literature [77,78]. We searched for articles published between 2000 and 2024 in the following databases: ScienceDirect, Scopus, Wiley, and Web of Science. We used the following search: Neuroprotective effects AND butyrate AND (Parkinson disease OR motor symptoms). 

### 4.3. Search Strategy

Four authors independently conducted the literature search in April 2024 (J.M.-B., E.M.O.-A., A.B.-G., and A.I.B.-V.), including the initial review of titles and abstracts and the evaluation of retrievable articles for comprehensive review. Included are original research studies written in English and a free full text.

### 4.4. Inclusion Criteria

The inclusion criteria required were as follows: (i) the relationship between intervention targeting butyrate regulation and changes in PD motor symptomatology as well as the neuroprotective effects was investigated, and (ii) the use of modulation of butyrate levels as a therapeutic target in PD motor symptomatology was evaluated.

The design of the studies consulted did not impose restrictions, thus encompassing both intervention and control groups. The following papers were excluded: Short Communication/Conference abstracts/Reviews/Mini Reviews. The search included original articles published between 2000 and 2024, with the sole criterion being that they were written in English.

## 5. Conclusions

Butyrate, particularly in its sodium butyrate (NaB) form, is emerging as a prominent neuroprotective agent in Parkinson’s Disease. Its ability to improve motor function, protect dopaminergic neurons, and reduce neuroinflammation positions NaB as a potentially effective therapeutic intervention in mitigating the pathological processes of PD. Beyond its effect on glutamate regulation, NaB demonstrates broad-spectrum action by influencing key pathways associated with inflammation and apoptosis, reinforcing its role as an integral modulator in the treatment of this pathology.

The singularity of NaB lies in its ability to act on multiple pathological fronts, suggesting that its inclusion in therapeutic strategies could offer significant benefits in disease management. Furthermore, the role of NaB in modulating the gut–brain axis opens new possibilities for interventions that integrate metabolic and anti-inflammatory aspects. Thus, these findings support the need to further explore the clinical potential of NaB in future studies with the aim of its possible implementation in innovative therapies for Parkinson’s patients.

In summary, NaB is emerging as a multifaceted compound with a broad spectrum of mechanisms of action, making it a robust candidate for future clinical investigation aimed at slowing PD progression and improving patient quality of life.

## Data Availability

Not applicable.
